# Application of an Acellular Dermal Matrix Allograft (CenoDerm) for Treatment of Multiple Gingival Recession Defects: A Case Report with One-year Follow-up

**DOI:** 10.5681/joddd.2014.033

**Published:** 2014-09-17

**Authors:** Neda Moslemi, Mohadeseh Heidari, Mahvash Mousavi Jazi, Mahdieh Daneshmonfared

**Affiliations:** ^1^Laser Research Center of Dentistry, Tehran University of Medical Sciences, Tehran, Iran; ^2^Associate Professor, Department of Periodontics , School of Dentistry, Tehran University of Medical Sciences, Tehran, Iran; ^3^Assistant Professor, Department of Periodontics, School of Dentistry, Mazandaran University of Medical Sciences, Mazandaran, Iran; ^4^Assistant Professor, Department of Periodontics, School of Dentistry, Tehran University of Medical Sciences, Tehran, Iran; ^5^Assistant Professor, Department of Periodontics, School of Dentistry, Shiraz University of Medical Sciences, Shiraz, Iran

**Keywords:** Acellular dermis, connective tissue, gingival recession

## Abstract

Several techniques and materials have been introduced for the treatment of gingival recession defects. This article reports the case of a 43-year old female patient with chief complaint of esthetic problem, presenting multiple gingival recession defects in anterior maxilla. CenoDerm combined with coronally positioned flap was used for management of six teeth with gingival recession. Complete root coverage was achieved in 66.6% of treated sites in one-year follow-up and the patient was satisfied with the esthetic result. The mean root coverage was 86.0%±22.3. The mean recession depth reduction and clinical attachment gain were 1.8±0.8 mm and 2.5±0.6 mm, respectively. According to the results obtained in this case, CenoDerm can be applied successfully in treatment of multiple gingival recession defects.

## Introduction

Gingival recession is a term used to describe apical displacement of gingival margin from the cemento-enamel junction (CEJ) that leads to exposure of root surface to the oral environment.^1^ Gingival recession may cause tooth sensitivity, tooth abrasion, root caries, and esthetic problems for the patient.^1,2^ Tissue trauma caused by vigorous tooth brushing is considered as the main etiologic factor for gingival recession.^2,3^

Complete coverage of recession defects with the appearance similar to surrounding tissue and minimal pocket depth is the ideal result of root coverage procedures.^2^ Numerous therapeutic approaches have been applied for root coverage in patients with gingival recession (e.g., laterally positioned flap, coronally positioned flap [CPF], subepithelial connective tissue graft [SCTG], CPF with matrix grafts, enamel matrix derivatives, and guided tissue regeneration [GTR]).^1,2^ According to previous meta-analysis study, using SCTG, matrix grafts, and enamel matrix derivatives would be superior to the application of CPF alone in achieving a complete root coverage.^4^ Due to significant patient-related limitations of SCTG technique, previous studies have focused mostly on non-autogenus grafts, especially when multiple sites need to be treated.^2,4,5,6^

Acellular dermal matrix allografts (ADMA—Alloderm; Life cell, The Woodlands, TX, USA) with CPF have shown similar results to SCTG in the treatment of gingival recession defects.^6,7^Application of ADMA can alternate the method of harvesting autogenous connective tissue graft.^1,6,7^ ADMA is a product of human skin that was initially used in plastic surgery in treatment of skin burn wounds. This matrix acts as a scaffold for the ingrowth of the host tissues.^5^

CenoDerm (Tissue Regeneration Corporation, Kish, Iran) is a new ADMA. It is a sterile freeze-dried dermal allograft in which cell components are removed and extracellular matrix consisting of collagen and elastic fibers is preserved. According to its manufacturer, it can be used for regenerative purposes such as gingival augmentation and root coverage. The present article reports a clinical case with one-year follow-up, employing CenoDerm with coronally positioned flap surgery to treat multiple gingival recession defects.

## Case Report 

**Figure 1.**
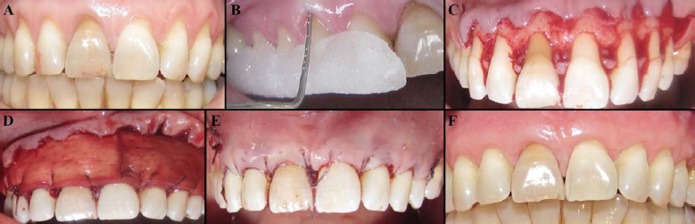
(A) Multiple gingival recessions in anterior maxilla; (B) measurement of the defects using an acrylic stent; (C) A tension free flap has been elevated; (D) CenoDerm has been placed and sutured with a resorbable suture; (E) continuous sling mattress suture; (F) One-year follow-up.

A 43-year old female patient with chief complaint of esthetic problem was referred to the Department of Periodontics at Tehran University of Medical Sciences. The patient had no systemic problem and was not smoker. There were multiple gingival recession defects in the area of teeth number 6 to 11 (Figure 1A). The affected teeth were without cervical caries or restorations. After taking informed consent, scaling, root planing, and polishing were performed and a non-traumatizing tooth brushing technique (roll method) with a soft toothbrush was instructed to the patient.

After injection of local anesthesia, root planing was gently performed for the exposed parts of the roots using a universal curette (McCall 17/18, Hu-Friedy, Chicago, USA) to obtain a hard and smooth root surface followed by a thorough rinse with the sterile normal saline. An intra-sulcular incision was made with number 15 scalpel blade (IsoMED; Wuxi Xinda Medical Device Co Ltd, Zhangjing Wuxi, China) between distal aspects of the maxillary first premolars. Two oblique releasing incisions were made at each end of the area, preserving the tips of the papillae. A full thickness flap was elevated to the mucogingival line. Then, an incision was made in the periosteum to create a partial thickness flap and to ensure the tension-free coronal displacement (Figure 1C).

CenoDerm (size: 2×2 cm, thickness: 2 mm; Tissue Regeneration Corporation, Kish, Iran) was soaked in a dish contained 50-100 cc sterile saline for 10 minutes. Then, it was trimmed to fit the dimensions of the defects. CenoDerm was placed over the exposed root surfaces, approximately 1 mm coronal to the CEJ, covering 3 mm of surrounding bone. The graft was fixed with continuous sling suture using a 4-0 resorbable polyglycolate coated suture material (Hur-Teb, Qazvin, Iran; Figure 1D). Then, the overlying flap was positioned coronally, completely covering the CenoDerm, and sutured without tension using continuous sling suture with the same suture material (Figure 1E).

The patient was instructed to take non-steroidal anti-inflammatory drug (Gelofen, 400 mg, per pain; Gelofen, Daana Pharma, Tabriz, Iran) and to use of 0.2% chlorhexidine gluconate mouthrinse (Hexodine, Donyaye Behdasht, Tehran, Iran) twice daily for two weeks. Sutures were removed after two weeks.

Patient was asked to avoid mechanical plaque control of surgical site for one month and then return to normal tooth cleaning using roll technique with a soft toothbrush. Patient was visited at two-week intervals.

Patient satisfaction was measured by asking the patient about the esthetic results of surgery and scoring from zero to 10. Clinical parameters were measured at baseline, and three, six, and 12 months after surgery. In order to assure reproducible measurements, an acrylic stent was used during measurement of clinical parameters (Figure 1B). All clinical parameters were measured by a standard Williams periodontal probe (Hu-Friedy, Chicago, USA). On each tooth, the middle part of the buccal surface was considered as the reference point and was demarcated on the acrylic stent. Clinical parameters were measured from the reference points. The measured values were rounded to the nearest 0.5 mm.

The following clinical parameters were measured:

- Probing pocket depth (PPD): Distance from margin of gingiva to sulcus (pocket) depth.
- Clinical attachment level (CAL): Distance from CEJ to sulcus depth.
- Recession depth (RD): Distance from CEJ to most apical part of the gingival margin.
- Recession width (RW): Mesial to distal width of recession at CEJ.
- Width of attached gingiva (AG): Distance from sulcus depth to mucogingival junction (determined with rolling the alveolar mucosa coronally with the side of a probe).^7,10^
- Mean root coverage: Percentage of changes of RD related to the baseline value.
- Complete root coverage: Percentage of teeth that showed 100% root coverage.

The healing phase was uneventful and patient did not report discomfort or pain along the healing period. Patient was satisfied in terms of esthetic results (nine out of ten). In addition, both color match and tissue contour were favorable.

The clinical measurements of recession depth at baseline and final visit are presented in Table 1. Of the six teeth treated, four teeth showed complete root coverage (66.6%). According to Wilcoxon signed ranks test, after one year, the clinical parameters improved significantly compared to baseline values (P < 0.05), except for the width of attached gingival (P = 0.083). Mean ± SD of the parameters of PD, CAL, RW, and attached gingiva at baseline and follow-up visits are given in Table 2.

**Table 1:** Initial and one-year recession depth measurements

Tooth No.	Initial recession (mm)	Final recession (mm)	Root coverage (%)
6	2	0	100
7	1	0	100
8	2	0	100
9	2	1	50
10	3	0	100
11	3	1	66.6
Mean ± SD	2.2 ± 0.8	0.3 ± 0.5	86.0 ± 22.3

**Table 2:** Clinical parameters of probing depth (PD), clinical attachment level (CAL), recession width (RW), and attached gingiva (AG) at baseline and 3, 6, and 12 months after root coverage procedure

Assessment	PD	CAL	RW	AG
Baseline	1.8 ± 0.4	4.0 ± 0.9	3.6 ± 0.5	1.8 ± 0.8
3 months	1.8 ± 0.4	2.2 ± 0.8	0.8 ± 1.3	2.8 ± 0.8
6 months	1.5 ± 0.5	1.8 ± 0.8	0.8 ± 1.3	2.3 ± 0.8
12 months	1.2 ± 0.4	1.5 ± 0.5	0.8 ± 1.3	2.3 ± 0.8

## Discussion

The present case showed satisfactory outcomes with CenoDerm used as an alternative graft to autogenous connective tissue for root coverage procedure without any adverse effects. The application of CenoDerm as a barrier membrane for GTR/GBR procedures have been suggested by the manufacturer and this is the first report regarding applicability of CenoDerm for root coverage procedures.

ADMA might have several advantages over SCTG for treatment of gingival recession defects. ADMA is commercially available in several sizes and is especially helpful when multiple sites are involved. Another advantage of this technique is that there is no need for a second surgical site for harvesting the graft, that results in less morbidity, post-surgical trauma, and discomfort for the patient. Therefore, the operatory time would be reduced and acceptance of the procedure by the patient would be improved.^5^

No study to date has evaluated the use of CenoDerm as an alternative to connective tissue graft for root coverage procedure. Therefore, comparison of the results of this case with other similar studies is not possible at present. However, considering Alloderm (Life cell, The Woodlands, TX, USA) as a material with potentially similar characteristics to CenoDerm, the clinical results of these two materials would be comparable.^6,7,8^ Cortes et al^8^ used AlloDerm in their study and reported 76% (2.6 mm) root coverage after 6 months, that is comparable to the results of the present case, which showed 86% (1.8 mm) root coverage with CenoDerm.

With regards to complete root coverage in this case, two teeth (teeth no. 9 and 11) did not show complete root coverage. The mild rotation of tooth no. 9 and protrusion of tooth no. 11 may explain this result.^9^

The authors used the thickest available CenoDerm that is comparable with that of autogenous connective tissue graft. The rationale behind this decision was that increasing the gingival thickness creates more robust marginal tissues, which might be more resistant to plaque induced inflammation or trauma and subsequent gingival recession.^6,8^

With regards to gain of attached gingiva, the result of this case is in accordance with those of the previous reports using Alloderm.^7,8^ The results of a systematic review showed there was no significant difference between CPF + Alloderm versus CPF alone in terms of keratinized tissue gain.^2^ Moreover, SCTG had more keratinized tissue gain than other methods.^2^ It was reported that the viability of the connective tissue might be necessary for a graft to induce keratinization of the overlying epithelium.^10^ Assuming that CenoDerm has similar characteristics to those of Alloderm, this finding is not unexpected. Therefore, in cases in which gain of keratinized tissue is one of therapeutic objectives, using allografts might not be the method of choice.

Although the improved parameters remained stable during one-year follow-up, the results obtained with CenoDerm in this case should be interpreted cautiously. Studies with large sample size are necessary to confirm the usefulness of the applied material and technique for root coverage.

## Aknowledgement

The authors declare no conflict of interests.
